# A perspective on photoperiodic phloem-mobile signals that control development

**DOI:** 10.3389/fpls.2013.00295

**Published:** 2013-08-02

**Authors:** David J. Hannapel

**Affiliations:** Plant Biology Major, Iowa State UniversityAmes, IA, USA

**Keywords:** FLOWERING LOCUST, mobile RNAs, potato, StBEL5, SP6A

## Abstract

Phloem-mobile signals that are regulated by day length activate both flowering and tuber formation. Both signaling processes have numerous elements in common. In this review, FLOWERING LOCUS T and the three signals currently implicated in controlling tuberization, SP6A, miR172, and the StBEL5 complex, are discussed with a focus on their functional roles, their mechanisms of long-distance transport, and their possible interactions.

## INTRODUCTION

Day length is critical in plants as an environmental cue for regulating numerous developmental processes. Recent reviews have addressed the remarkable similarities between photoperiodic signaling in both flowering and tuberization (Suárez-López, 2005; Rodríguez-Falcón et al., 2006; Abelenda et al., 2011). Both involve phloem-mobile signals with the best example being FLOWERING LOCUS T (FT; reviewed by Turck et al., 2008). Under inductive conditions, the B-box zinc finger protein CONSTANS (CO) induces transcription of FT in the phloem. The FT protein then moves through the sieve element system into the shoot apex where it interacts with the bZIP transcription factor (TF), FLOWERING LOCUS D (FD), to activate the floral pathway. Several studies have identified FT in the shoot apex or phloem exudate of plants induced for flowering (Corbesier et al., 2007; Lin et al., 2007; Tamaki et al., 2007; Yoo et al., 2013). FT is a member of a family of proteins that contain a phosphatidylethanolamine-binding domain (PEBP) and is not itself a TF (Kardailsky et al., 1999; Kobayashi et al., 1999). FT acts as a co-regulator of FD to facilitate binding to floral identity genes like *APETALA1*. In this way, FD provides spatial control of flowering and FT provides temporal control. Recent studies suggest that an anti-florigenic signal may also be trafficking long distance. This *CEN/TFL1* homolog, designated *ATC*, is expressed in the phloem and not the shoot apex. Genetic analysis showed that *ATC* suppresses flowering and that both its mRNA and protein can move through a graft junction (Huang et al., 2012). After entering the shoot apex, ATC may then compete with FT for binding to FD. So what can we learn from flowering that will help us better understand the phloem-mobile signal that regulates tuberization?

The history of the mobile signal of potato is based on the work of numerous physiologists that have pieced together the story over several decades (Bernard, 1902; Garner and Allard, 1920, 1923; Gregory, 1956; Chapman, 1958; Kumar and Wareing, 1973). They showed that under conditions of low temperature and short days (SDs), a graft-transmissible signal produced in leaves moves down the phloem system into stolons to induce tuberization. Using a tobacco/potato heterograft, the floral signal from a tobacco scion can induce tuberization in a non-induced potato stock (Chailakhyan et al., 1981). Clearly, the signaling process of both flowering and tuberization share common themes. The advent of genome sequence and extremely sensitive molecular detection methods have made it possible to identify several potential signals involved in the process. As potential standards, let us consider some experimental criteria for assessing the functional role of a putative phloem-mobile tuberization signal. (1) If it is phloem-mobile then it should be detected in phloem cells or sap and it should transverse a heterograft. Of course, this presents detection issues for specific agents and is a limiting factor with some prime candidates. (2) Because movement may be technically difficult to confirm, then at least, accumulation of the signal should be associated in someway with tuber initiation or development. Over-expression or suppression should affect tuber development or morphology. Over-expression should be able to overcome the negative effects of long days (LDs). Certainly a knock-out mutant would be advantageous but redundancy is very likely built into this important biological process. (3) And finally, some component of the complex should be photoperiod regulated, verifying that the leaf is the origin of the activator or repressor signal. The three best candidates for phloem-mobility signals that regulate tuber formation and will be discussed in this perspective are StFT/SP6A, miR172, and the *StBEL5* RNA complex. Other candidate signals will likely emerge in the near future, which would add to the growing notion that there is redundancy in phloem-mobile tuberization signals. Some of these signals may also function as components in flowering pathways. This review discusses the current evidence for phloem-mobile signals controlling tuberization and/or flowering.

## StSP6A

It is now readily apparent that FT-like genes function in a wide range of developmental events beyond flowering (Pin and Nilsson, 2012). Consistent with a role for StFT/SP6A as the tuber signal, transgenic over-expression lines tuberized under non-inductive LDs, whereas transgenic suppression lines exhibited a strong reduction in tuber production under SDs (Navarro et al., 2011). Local induction of *StSP6A* transcripts in stolons activated several tuber-identity genes including *StGA2ox1* (Navarro et al., 2011). Potato StCO which has a negative effect on tuberization (González-Schain et al., 2012) also represses *StSP6A* gene expression under LDs. Whereas StSP6Aox scions grafted onto wild-type stocks induced the stocks to tuberize, there was no detection of StSP6A protein moving through the graft unions. This could be due to technical limitations. More support for activity by phloem-mobile FT proteins came from the demonstration that the rice FT orthologue Hd3a fused to GFP can move through a graft into a stolon (Navarro et al., 2011). Supporting the common theme with flowering, this Hd3a construct was able to increase tuber production in over-expression lines under LDs as well as through heterografts with wild type (WT) stocks (Navarro et al., 2011). Hd3a functions in a hexameric floral activation complex composed of three homodimers of OsFT, OsFD and a 14-3-3 protein that functions as a scaffold (Taoka et al., 2011).

The data available on StSP6A strongly implies it is a very likely candidate for a mobile tuber signal. Despite the evidence for StSP6A as a tuber-inducing signal, several questions remain to be answered. How is StSP6A gene expression induced in leaves under SDs? If the protein moves from leaf to stolon, why does *StSP6A* RNA accumulate in stolons in response to SDs? What is the mechanism for StSP6A LD repression in leaves? Is it by StCO activity as was previously assumed or by StSP5G competition in a mechanism similar to the antagonistic interplay between FT-orthologs of sugar beet that respectively promote or suppress flowering (Pin et al., 2010)? In this system, one sugar beet FT protein is essential for flowering whereas the other suppresses it. Finally, StSP6A is also a member of the PEBP family and does not act alone. As a co-regulator, StSP6A will likely form a dimer with a functional TF like FD. If so, what is the identity of such a transcription factor and how does it mediate StSP6A activity and tuber-specific gene expression?

## miR172

Movement of miR172 represents a unique and interesting aspect of regulation in the tuberization system. The processing of miR172 is known to be mediated by GIGANTEA and it is involved in the photoperiodic control of flowering (Jung et al., 2007). Over-expression of this microRNA in potato promotes flowering and activates tuber formation under LDs (Martin et al., 2009). Although no movement of miR172 was detected, the presence of this microRNA could be detected in the vascular bundles and its effect on tuberization was graft transmissible. A model was proposed wherein miR172 acts downstream of the tuberization repressor phytochrome B and upstream of the tuberization activator StBEL5. As a hint to function, a miR172 binding site was identified in an APETALA2-like mRNA, *RAP1*, which was downregulated in a phytochrome B antisense line. In this model, miR172 induces the degradation of *RAP1* which may then influence *StBEL5* expression. Because of its role in suppressing translation and enhancing degradation of target RNAs, it is difficult to separate direct movement of miR172 and a localized function in stolons from repression (via transcript degradation) of the movement of one of its targets that may influence tuberization. Overall, these results suggest, however, that miR172 plays important roles in regulating both flowering and tuber induction in potato.

## THE StBEL5 RNA COMPLEX

BEL1-like transcription factors function by binding to KNOTTED1-types (Hamant and Pautot, 2010). These two ubiquitous families regulate a number of pathways controlling hormone synthesis and signaling in plants (Bolduc et al., 2012). There is considerable information available on the transcriptional role of StBEL5 and one of its KNOTTED1 partners, POTH1, and their putative role as mobile signals (Chen et al., 2003, 2004; Banerjee et al., 2006, 2009; Mahajan et al., 2012). Movement and accumulation of *StBEL5* RNA have been consistently associated with enhanced tuberization even under LDs. But StBEL5 also increases earliness (initiation) in tissue culture plants under both LD and SD conditions (Chen et al., 2003). *StBEL5* RNA has been detected in phloem cells using three different approaches: *in situ* hybridization and RT-PCR of phloem sap and RNA extracted from phloem cells harvested by using laser capture microdissection (Banerjee et al., 2006; Yu et al., 2007; Campbell et al., 2008).

**FIGURE 1:**
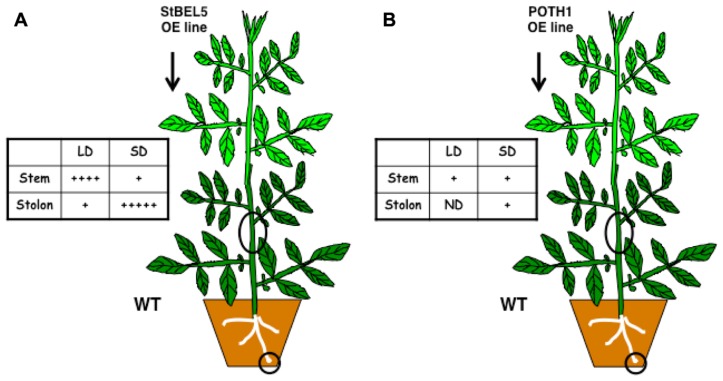
**Full-length *StBEL5* (A) and *POTH1* (B) RNA movement in a downward direction in response to photoperiod in soil-grown heterografts.** Both RNAs were quantified using gene-specific primers that amplified only transgenic RNA in real-time qRT-PCR and their relative abundances are shown with a (+) sign. WT sample sources are indicated by black circles. The transgenic lines expressed full- length *StBEL5* or *POTH1* RNA driven by the 35S CaMV promoter. Grafts were wrapped in plastic and allowed to take for 2 weeks at 25°C under long-day conditions and were then transferred to short days (SDs) or maintained under long days (LDs) for two more weeks. Upward movement in a WT/transgenic heterograft was also observed for *StBEL5* into stem and leaves and into stem for *POTH1*. ND, not detected. Data from **(A)** was used with permission of the author (Hannapel, 2013).

Movement into stolons was confirmed in heterografts and in two transgenic whole plant systems with two different promoters (Banerjee et al., 2006; **Figure 1**). The use of transgenic over-expression lines with non-plant sequence tags has been critical in establishing movement assays and clarifying the role of untranslated regions (UTRs) in this process. Without such an approach, it would be impossible to detect mobility or to distinguish endogenous *StBEL5* RNA from transgenic. The source of *StBEL5* RNA has also been clearly established providing further insight on the mechanism of its mobility. For example, despite the observation that there are copious amounts of *StBEL5* transcripts in the stem of WT plants, promoter activity is essentially absent in this organ (Banerjee et al., 2006). Both *POTH1* and *StBEL5* RNAs move freely throughout the plant with a concentration of *StBEL5* transcripts in SD stolons (**Figure 1**; Hannapel, 2013).

Movement into stolons is regulated by photoperiod and enhanced by the UTRs of *StBEL5*. *StBEL5* UTRs were fused to another RNA, *StBEL14*, to make it more mobile (Banerjee et al., 2009). This directed accumulation of this non-mobile *StBEL* RNA was correlated with enhanced yields. SD induction of *StBEL5* promoter activity in dark-grown stolons has been observed (Chatterjee et al., 2007) and explained by a mechanism of auto-regulation (Lin et al., 2013). Such auto-regulation by a RNA induced to move by short-day conditions is a classic example of light transduction to an underground organ. Several potential targets of the StBEL5 protein have been identified and specific tandem TTGAC target elements have been confirmed by gel-shift analysis (Hannapel et al., 2013; Lin et al., 2013). These include *GA2ox1*, *ISOPENTENYL TRANSFERASE*, *YUCCA1* and several other genes involved in hormone metabolism. Putative protein chaperones that may facilitate *StBEL5* movement and stability have been identified (Cho et al., 2012; Mahajan et al., 2012).

Despite the fact that neither protein has been detected in phloem cells, one cannot rule out the possibility that StBEL5 and/or POTH1 proteins act as long-distance signals in this developmental process. In addition, StBEL5 and POTH1 are not specific for tuberization. *StBEL5* is mobile to roots where it influences development (Lin et al., 2013). POTH1 appears to function in leaf structure and overall plant architecture (Rosin et al., 2003). *StBEL5* RNA is ubiquitous and even present in stolons from plants cultivated under LDs. Although lower in abundance, RNA of *POTH1* is also ubiquitous, which contradicts the need for *StBEL5* and *POTH1* RNAs to function as phloem-mobile tuberization signals. However, from animal systems it is known that RNA-binding proteins not only facilitate intracellular localization, but they can also contribute to repression of translation (St. Johnston et al., 1991; Colegrove-Otero et al., 2005; Shen et al., 2009). In many cases, these mobile RNAs are key transcription factors, and it is critical that their translation occur in the targeted tissues (King et al., 2005). Hence, specificity of phloem-mobile RNAs might be incurred by translational repression of RNA-binding proteins in non-target tissues. As examples, the UTRs of both *POTH1* and *StBEL5* suppress translation and these UTRs both bind to specific RNA-binding proteins (Banerjee et al., 2009; Mahajan et al., 2012).

It must be made clear, however, that there is no direct evidence that mobile *StBEL5* and *POTH1* RNA are required for tuberization. In this regard, however, one must consider the possibility of redundancy. There are several lines of information that suggest this possibility. Early antisense lines of both BEL5 and POTH1 showed no phenotype. There are 13 potato BEL genes and all have almost identical conserved functional regions, i.e, the BELL domain and the homeodomain. At least six *StBEL* genes show SD-induced accumulation of their RNAs in stolons. Besides *StBEL5*, the most promising candidates are *StBEL11* and *-29*. Both are detected in phloem sap and microdissected phloem cells, both are closely related phylogenetically to StBEL5, and all three are strongly induced by SDs in stolons. Together these three StBELs make up 71% of all potato BEL transcripts in the plant (The Potato Genome Sequencing Consortium, 2011). *StBEL5*, *-11*, and *-29* are also very abundant in petioles, a key organ for transporting RNAs into the stem. Redundancy was actually simulated by fusing *StBEL5* UTRs onto *StBEL14* and making it mobile to stolons where it enhanced tuber growth (Banerjee et al., 2009). The same rationale for redundancy also exists for the potato KNOX family. There are three other StKN1-type transcription factors that exhibit greater levels of RNA in phloem cells than POTH1 (unpublished RNA-Seq data). Any of these are potentially mobile and could act in direct interaction with StBEL5. In addition to POTH1, there are two other reports of KN1-type mRNAs that are phloem mobile (Kim et al., 2001; Ham et al., 2009).

## CONCLUSION: DO PHLOEM-MOBILE SIGNALS HAVE OVERLAPPING FUNCTIONS?

It is conceivable that there might be more than one pathway leading to tuber formation. There are five major pathways controlling flowering time in *Arabidopsis* (Yamaguchi and Abe, 2012) and each is adapted to respond to different environmental conditions. To ensure efficiency, genetic control of these pathways is mediated by regulatory “hubs” like FLOWERING LOCUS C, FT, SUPRESSOR OF OVEREXPRESSION OF CO1 and LEAFY. Formation of a tuber represents a similar substantial investment in photosynthate and is a very costly bioenergetic process that may also be regulated by such “hub” genes. It is feasible that overlap in function may occur among StSP6A, StFD, miR172, and StBEL5 and that back-ups are in effect to ensure that the process of tuberization is initiated and completed efficiently. This could explain the rationale for the increase of *StSP6A* RNA and auto-regulation of *StBEL5* in stolons.

Is it significant that *GA2 oxidase1* is a downstream target of both StBEL5 and StSP6A? StGA2ox1 plays a critical role during tuber formation by reducing GA levels in the stolon tip and transcript levels increase more than 70-fold at the onset of tuberization (Kloosterman et al., 2007). What elements are present in upstream sequence of *StGA2ox1* that may provide insights as to the regulator that controls its expression? Of the eight tuber-identity genes that are induced by StSP6A (Navarro et al., 2011), all eight contain tandem TTGAC elements in their upstream sequences and are likely target candidates of a StBEL5 complex. *StGA2ox1* contains five tandem TTGAC elements present in the first intron and upstream sequence of its gene including two tandem motifs 85 nucleotides apart, both containing TGAC elements on opposite strands two nucleotides apart that form a palindrome (Lin et al., 2013). The maize ortholog of *GA2ox1* also contains a tandem TTGAC element in its first intron (Bolduc and Hake, 2009), suggesting conservation of this transcriptional complex across species. The fact that *StGA2ox1* and other tuber genes may be regulated by both StBEL5/KNOX and StSP6A complexes implies cross-talk or direct interaction between these regulatory pathways. In planning future research, let us consider that we need to know more about miR172 targets, that StSP6A requires a transcription partner to make it a factor in expression, and that tuber-specific activity of *StBEL5* may require an additional co-regulator and post-transcriptional control mechanisms to allow for targeted movement, enhanced stability, and translational repression.

## Conflict of Interest Statement

The author declares that the research was conducted in the absence of any commercial or financial relationships that could be construed as a potential conflict of interest.
